# "The Hospital" Library and Charities Bureau

**Published:** 1903-12-12

**Authors:** 


### "THE HOSPITAL" LIBRARY AND CHARITIES BUREAU.

The attention which has been recently so generally drawn to the subject of hospital planning gives us an opportunity of bringing to the notice of our readers facilities offered by the above institution to those desirous of studying any aspect of the hospital question. In the library is to be found all the principal literature on the subject, and the largest collection existing of plans of hospitals all over the world. The library is comfortable and suitably furnished, so that those coming to study are conveniently accommodated, and the librarian is always glad to assist visitors in securing the information they require. Through the bureau questions can be answered by post. There is a small yearly subscription, and a still smaller fee for an isolated visit to the library. The library is open from 12 to 4 P.M. on week-days, Saturday excepted, and all information can be obtained from the Librarian, "The Hospital" Library and Charities Bureau, 29 Southampton Street, Strand, W.C.

